# Kinetic models of quality parameters of spreadable processed Gouda cheese during storage

**DOI:** 10.1007/s10068-018-0377-2

**Published:** 2018-05-15

**Authors:** Dorota Weiss, Jerzy Stangierski, Hanna Maria Baranowska, Ryszard Rezler

**Affiliations:** 1 Department of Food Quality and Safety Management, Faculty of Food Science and Nutrition, Poznań University of Life Sciences, Wojska Polskiego 31/33, 60-624 Poznan, Poland; 2 Department of Physics and Biophysics, Faculty of Food Science and Nutrition, Poznań University of Life Sciences, Wojska Polskiego 38/42, 60-624 Poznan, Poland

**Keywords:** Processed cheese, Shelf-life modelling, Kinetic models, Arrhenius equation

## Abstract

The aim of the study was to prepare mathematical models based on the Arrhenius equation as predictive tools for the assessment of changes in quality parameters during the storage of spreadable Gouda cheese at temperatures of 8, 20 and 30 °C. The activation energy value and the chemical reaction rate constant enabled the construction of kinetic models, which helped to estimate the direction and rate of changes. Moreover, the activation energy (E_a_) of the quality parameters was used to determine the sequence of their vulnerability during storage. The value of activation energy corresponding to temperature changes resulted in the following order of susceptibility of the quality parameters: ΔC > ΔE ≈ water activity > texture parameters > pH > colour > sensory parameters > rheological parameters. The research showed limited applicability of the mathematical models for estimation of quality parameters referring to spreadable processed Gouda cheese.

## Introduction

For more than one hundred years processed cheese has been a well-known food item produced in the processes of pasteurisation or sterilisation. The stability of the sensory quality and physicochemical characteristics of a product depends on various factors, such as the state and type of raw materials used for its production, the technological process, the type of package and the microbiological state of a ready product. As far as processed cheese is concerned, during storage we can observe major changes in its aroma and flavour (BK Giulini GmbH-BU-FOOD/BL-DAIRY, 2012; Singh et al., 2009), colour (Kapoor and Metzger, 2008; Weiss et al., 2015) and consistency (Buňka et al., 2008).

The shell-life given on packages by food producers is based on their knowledge and the results of tests, but they are not always fully consistent. Food science successfully and broadly uses the Arrhenius equation for the modelling of time and temperature dependences. Singh et al. (2009) proposed a model of sensory changes in UHT milk based on selected parameters referring to progressing unfavourable changes during storage, such as browning, oxidation and lipolysis. Calligaris et al. (2004) used this dependence to study the kinetics of oxidative changes in sunflower oil at temperatures ranging from − 30 to 60 °C. The Arrhenius equation has also been used for the modelling of changes in selected features of food products resulting from exposure to high and low temperatures (Zanoni et al., 2007) and for the specific rate of growth of the microorganisms which cause the rotting of minced beef (Limbo et al., 2009).

Available publications give numerous examples of various mathematical models which were developed to assess the prediction of changes occurring during the storage of e.g. dairy foods, including some yoghurt (Zhi et al., 2018). Many models were developed as a result of research on plant products: modelling of the effect of storage temperature on the respiration rate and texture of fresh cut pineapple (Benítez et al., 2012), Weibull hazard analysis for estimation of the shelf-life of pezik pickles (Keklik et al., 2017), kinetics of changes in shelf-life parameters during storage of pearl millet (Bunkar et al., 2014), non-isothermal kinetic modelling for anthocyanins in bread and crust (Sui et al., 2015), prediction of the quality and storage period of soybean at different temperatures (Dong et al., 2015). The fast mathematical modelling methods such as the Weibull hazard model and Q10 model have been used with meat products to predict the shelf-life of chilled pork (Tang et al., 2013), for the modelling of frozen shrimp shelf-life at variable temperatures (Tsironi et al., 2009), the kinetics of changes in the quality of *Pangasius* fillets at stable and dynamic temperatures, and to simulate downstream cold chain conditions (Mai and Huynh, 2017).

In order to determine the dynamics of changes it is necessary to use the mathematical equation which will enable the identification and consideration of a factor or group of factors responsible for quality loss and changes in quality parameters. Temperature and storage time are the external factors that have the greatest influence on the deterioration of spreadable processed Gouda cheese during cold chain storage. The influence of other parameters, especially the ones associated with the external environment, is limited because the package protects the product from the direct influence of environmental conditions, such as light and air (Bao et al., 2013).

There are no publications with data describing changes in such a big group of physicochemical and sensory factors during the storage of processed cheese at different temperatures. The aim of the study was to assess the usefulness of kinetic models based on the Arrhenius equation as predictive tools for the assessment of changes in quality parameters during the storage of spreadable Gouda cheese at different temperatures. The activation energy (E_a_) of quality parameters was used to determine the sequence of their vulnerability during storage.

## Materials and methods

### Processed cheese preparation

Samples of spreadable processed cheese were produced in an industrial setting as part of a batch. The following raw materials were used in this process: water, natural ripened cheese, milk proteins, butter, emulsifying salts and others. The selected ingredients were ground and weighed. Then, the raw materials were sequentially dosed to a cooker and melted at temperatures of 80–85 °C for 5 min. The technological process took place at the manufacturer’s factory. It resulted in a stable cheese mass, which was packed into plastic tubes of 140 g. Next, the products were cooled to a temperature of 30 °C in a cooler system for 40 min. Then, the samples were stored at three temperatures: 8, 20 and 30 °C (± 1 °C). The samples came from three different batches. They were stored under experimental conditions until the end of their shelf-life—for 120 days after the production or until the signs of product spoilage appeared. Spoiled samples exhibited significant changes in their consistency and colour as well as off-odours and gassing defects.

### Sample preparation

The samples underwent physicochemical analyses and sensory evaluation to find changes in their quality during storage under various conditions. On each day of the experiment a new pack of cheese was opened for analysis. The samples were analysed at a temperature of 20 ± 1 °C. The samples incubated at 8 and 20 °C were analysed once a week, whereas those incubated at 30 °C were analysed once a day. The samples were stored at 8, 20 and 30 °C for 120, 63 or 10 days after the production, respectively.

### pH measurement

Acidity was measured with a pH-meter with a combined glass electrode (FiveEasy, Mettler-Toledo). After each measurement the electrode was calibrated using a pH 7.0 buffer (Merck, Germany).

### Water activity measurement

Samples of 30-mm diameter and 10-mm thickness were prepared. A water diffusion and activity analyser ADA-7 (COBRABID Poznań, Poland) with a system of automatic registration of water evacuation from an individual sample was used for measurements. Before each measurement the chamber was dried to a water activity of 0.1.

### Water binding dynamics

Samples of 0.2 cm^3^ were placed in measuring tubes and sealed with Parafilm “M”^®^. An NMR Pulse PS 15T Spectrometer (Ellab Poznań, Poland), operating at a frequency of 15 MHz was used to measure spin–lattice (T_1_) and spin–spin (T_2_) relaxation times. The T_1_ relaxation time was measured with the pulse sequence (π–TI–π/2). The distance between the pulses (TI) varied from 1 to 800 ms and the repetition time was 15 s. 32 FID signals and 110 points for each FID signal were collected. Spin–spin (T_2_) was measured with the Carr-Purcell-Meiboom-Gill (CPMG) pulse train (Brosio and Gianferri, 2009). The spin–lattice and the spin–spin relaxation times enabled calculation of the average time correlation (τ_c_).

### Rheological properties

These measurements were taken with a Dynamic Mechanical Thermal Analysis (DMTA) spectrometer DMWT (COBRABID, Poznań, Poland) (Stangierski et al., 2014). A cone-plate measuring system was used. The component of the complex modulus of elasticity (G′), the loss modulus (G″), loss tangent (tgδ) and dynamic viscosity (η) were measured. G′ is associated with this part of potential deformation energy which is maintained in the course of periodical deformations. G″ reflects the portion of energy that is dissipated in the form of heat. The loss tangent (tgδ) is a measure of internal friction and refers to the relative quantity of energy dissipated in the material during one deformation cycle. The spectrometer made free vibration analysis in the inverted torsion pendulum, plate–cone at a vibration frequency of 2.1 Hz.

### Texture measurement

The texture of the samples was measured with a TA-XT plus Texture Analyser (Surrey, England) and TTC Spreadability RIG (HDP/SR) probe at an angle of 45°. The product was forced to flow outward at 45° between the male and female cone surfaces during the test, the ease of which indicated the degree of spreadability. Withdrawal of the cone probe from the sample provided information about possible adhesive characteristics. The probe moved at a speed of 2 mm/s over a distance of 23 mm. The following texture attributes were measured: spreadability (Nxs), hardness (N), adhesiveness (Nxs), and the viscosity index (N).

### Colour assessment

The colour changes were determined by measurement of Comission Internationale de l*’*Eclairage (CIE) L*, a*, b* values (L*—lightness, a*—redness, b*—yellowness) with a Minolta Chroma Meter CR 200 colorimeter (Osaka, Japan). The measuring head was 8 m in diameter. The device was equipped with a C light source and a standard 2° observer. This colorimeter was calibrated using a white standard (Y = 94.0, x = 0.3131, y = 0.3203).The colour change (ΔE) (1) and the chroma (ΔC) (2) were calculated as follows (Benítez et al., 2012):

1 $$\Delta E = \sqrt {\left( {L_{1}^{*} - L_{2}^{*} } \right)^{2} + \left( {a_{1}^{*} - a_{2}^{*} } \right)^{2} + \left( {b_{1}^{*} - b_{2}^{*} } \right)^{2} }$$

2 $$\Delta C = \sqrt {\left( {a_{1}^{*} - a_{2}^{*} } \right)^{2} + \left( {b_{1}^{*} - b_{2}^{*} } \right)^{2} }$$

where L_1_*, a_1_*, b_1_*—colour values of the white standard; L_2_*, a_2_*, b_2_*—colour of the sample under analysis.

### Sensory evaluation

An unstructured linear scale in the form of an 80 mm segment was used for sensory evaluation. The scale of intensity had border-value descriptions for each distinguishing feature under analysis, where 1 referred to a very undesirable quality, whereas 9 referred to a very desirable one. The following distinguishing features of cheese were evaluated: taste, aroma, texture, colour and general desirability. The evaluation panel consisted of 28 people, who had been trained and prepared for the task. Each member evaluated the product according to their individual impression, which corresponded to the product quality related with the specific feature. Before statistical analysis the results of the sensory evaluation parameters were converted into percentages.

### Statistical analyses

The samples were evaluated at least three times. The results were expressed as mean ± SD. The mean values were compared by means of Tukey’s post hoc test. The significance of variability factors to the measured effect was determined by means of analysis of variance (ANOVA). The statistical significance level was *p* < 0.05. The Statistica v.13 (StatSoft, Poland) and Excel Statistical software was used for statistical analyses.

### Modelling

The graphical method was used to determine the order of reaction for all quality parameters. Quality deterioration during storage was analysed by means of a linear regression in the following order of reaction: zero, first and second. The most accurate fit was confirmed by the determination coefficient—R^2^. At the next stage the Arrhenius equation parameters: activation energy (E_a_) and reaction rate constant (k) were calculated for all the quality characteristics of processed Gouda cheese. The logarithmic form of the Arrhenius equation was used for this purpose (1):

3 $${\text{lnk}} = {\text{lnA}} - \left( {\frac{{{\text{E}}_{\text{a}} }}{\text{RT}}} \right)$$

where k—reaction rate constant; A—pre-exponential factor; E_a_—activation energy [J/mol]; R—gas constant [8.314 J/K mol]; T—absolute temperature [K].

The relations between certain features and storage time for each order of reaction can be expressed with the following mathematical equations (Honga et al., 2012):

For the zero-order reaction, where n = 0:

4 $${\text{Q}} = {\text{Q}}_{0} {-}{\text{kt}}$$

For the first-order reaction, where n = 1:

5 $${\text{Q}} = {\text{Q}}_{0} \exp ({-}{\text{kt)}}$$

For the second-order reaction, where n = 2:

6 $$\frac{1}{\text{Q}} = \frac{1}{{{\text{Q}}_{0} }} + {\text{kt}}$$

The Arrhenius Eq. (3), activation energy value, reaction rate constant and Eqs. (4), (5) and (6) were used to prepare kinetic models of changes in the quality parameters during storage. The function of the quality of the zero-order reaction can be expressed as follows:

7 $${\text{Q}} = {\text{Q}}_{0} - k\exp \left( {\frac{{ - E_{a} }}{RT}} \right)$$

The model was verified with the determination coefficient R^2^ applied to the values of estimated and observed quality parameters. The Statistica v.13 (StatSoft, Poland) software was used for statistical calculations at a significance level of α = 0.05.

## Results and discussion

During the whole test there were minor changes in the quality parameters of the samples stored at 8 °C. It suggests that storage time did not have significant influence on the product. Tukey’s test showed no statistically significant differences in most of the parameters. The changes observed in the samples stored at 20 °C were dynamic and they resulted in a significant decrease in the product quality. However, the highest rate of changes in the quality parameters was observed in the samples stored at 30 °C. The changes were negative and led to the product decomposition. The results of the sensory analysis showed that taste, smell and consistency were the first parameters to exhibit changes in all the samples. The findings let us assume that the approximate shelf-life limit for spreadable processed cheese stored at 8, 20 and 30 °C without significant changes in its quality would be 49, 28 and 4 days, respectively.

The reaction rate referring to the quality parameters was evaluated by means of scatterplots and regression analyses. The majority of the parameters were characterised by the first- and zero-order reactions and only in a few cases—by the second order. The second-order reactions were characteristic of the following texture attributes: spreadability, viscosity index and adhesiveness. Table 1 shows the results of the reaction rate order. Changes in the food product quality are caused by physicochemical (Kapoor and Metzger, 2008) and microbiological reactions (BK Giulini GmbH-BU-FOOD/BL-DAIRY, 2012; Kapoor and Metzger, 2008). The rate of these reactions chiefly depends on the storage temperature. Changes in the processed cheese taste and aroma are caused by the aldehydes and ketones formed in the process of fat and protein oxidation (BK Giulini GmbH-BU-FOOD/BL-DAIRY, 2012; Sunesen et al., 2002). They are also caused by volatile compounds produced by vegetative and sporulating microorganisms (Varga, 2005). Due to the high complexity of the processes causing these changes in the quality of products during storage they are referred to as pseudo-first-order reactions (Spiess et al., 1998).

**Table 1:** The order of reaction referring to the quality attributes of processed Gouda cheese—estimation based on the determination coefficient (R^2^)

Quality parameter	Zero order reaction rate	First order reaction rate	Second order reaction rate
Water activity	37.12 ± 0.00	37.36 ± 0.00	38.23 ± 0.00
Acidity (pH)	37.47 ± 0.01	37.03 ± 0.01	37.49 ± 0.00
Lightness L*	31.63 ± 0.00	31.59 ± 0.00	31.60 ± 0.00
Redness a*	18.35 ± 0.01	17.57 ± 0.01	17.22 ± 0.00
Yellowness b*	21.91 ± 0.00	22.17 ± 0.01	22.11 ± 0.00
Difference in colour ΔE	37.60 ± 0.07	27.13 ± 0.18	7.64 ± 0.23
Difference in chroma ΔC	28.34 ± 0.07	18.37 ± 0.22	6.40 ± 0.37
Dynamic viscosity η	49.15 ± 0.00	48.71 ± 0.01	47.14 ± 0.00
Elastic modulus G′	17.96 ± 0.00	18.09 ± 0.00	18.12 ± 0.00
Loss modulus G″	50.63 ± 0.00	50.29 ± 0.00	49.07 ± 0.00
Loss tangent tgδ	15.44 ± 0.00	15.75 ± 0.01	14.02 ± 0.05
Hardness	20.74 ± 2.51	20.74 ± 4.52	8.97 ± 0.00
Spreadability	38.42 ± 0.01	42.29 ± 0.03	47.02 ± 0.00
Viscosity index	21.68 ± 0.01	23.64 ± 0.03	24.76 ± 0.00
Adhesiveness	45.32 ± 0.01	49.01 ± 0.03	53.80 ± 0.00
Colour	19.07 ± 0.00	18.90 ± 0.00	18.71 ± 0.00
Consistency	10.16 ± 0.00	9.52 ± 0.00	8.86 ± 0.00
Smell	76.48 ± 0.02	76.47 ± 0.13	73.79 ± 0.00
Taste	79.76 ± 2.96	42.02 ± 6.54	68.03 ± 0.00
Overall desirability	86.48 ± 0.01	87.06 ± 0.07	83.05 ± 0.00

The Arrhenius equation, expressed with the general formula (3), was used to calculate activation energy (E_a_) and the reaction rate constant (k_0_) for each quality parameter. Table 2 shows the results. The ΔC and ΔE parameters were the lowest values of activation energy among all colour parameters. This suggests that these features were the most sensitive to the changes in storage temperature. However, the reaction rate constant (k) was the highest for these parameters, which implies that the slowest rate of reaction occurred despite the small amount of energy necessary to trigger the change process. Hardness was another parameter with a low activation energy value but a high value of the reaction rate constant. The value of this factor suggests that hardness changed the fastest of all the textural parameters of this product. The rheological and sensory parameters were the most stable features of processed Gouda cheese due to the highest activation energy values. As results from the activation energy value influenced by changes in the temperature, the quality parameters were characterised by the following order of susceptibility: ΔC > ΔE ≈ water activity > texture parameters > pH > colour > sensory parameters > rheological parameters.

**Table 2:** Activation energy (E_a_) and reaction rate constant (k_0_) values—estimation based on the Arrhenius equation

Quality parameter	E_a_ (kJ/mol)	k_0_
Water activity	79.31	8.95 × 10^10^
Acidity (pH)	97.63	8.78 × 10^14^
Lightness L*	190.49	1.61 × 10^30^
Redness a*	132.40	2.67 × 10^21^
Yellowness b*	173.22	3.52 × 10^28^
Difference in colour ΔE	78.70	3.48 × 10^12^
Difference in chroma ΔC	29.69	2.59 × 10^3^
Dynamic viscosity η	402.80	1.09 × 10^71^
Elastic modulus G′	621.79	2.48 × 10^109^
Loss modulus G″	339.13	8.27 × 10^57^
Loss tangent tgδ	132.64	4.24 × 10^21^
Hardness	44.15	4.54 × 10^9^
Spreadability	85.22	5.78 × 10^12^
Viscosity index	95.47	5.02 × 10^14^
Adhesiveness	76.29	1.54 × 10^11^
Colour	154.71	1.34 × 10^24^
Consistency	300.14	1.55 × 10^51^
Smell	243.87	5.44 × 10^42^
Taste	111.45	1.72 × 10^20^
Overall desirability	236.29	1.20 × 10^41^

**Fig. 1:**
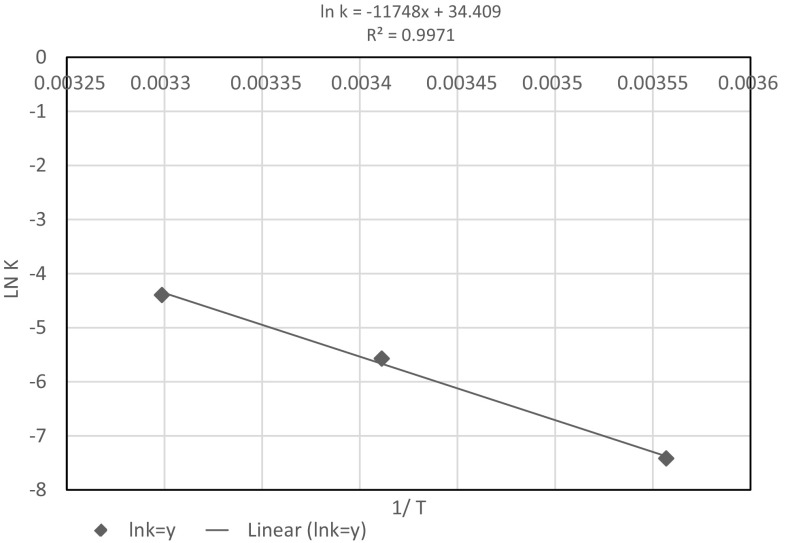
The regression equation used for calculation of k_0_ and e_a_ constants in the Arrhenius equation referring to the acidity of processed Gouda cheese

The reaction rate constant of the processed cheese quality parameters was calculated using linear regression analysis between ln k and 1/T. Figure 1 shows an example of the regression equation for acidity.

The values of the order of reaction, the activation energy and the rate constant were used to prepare kinetic models of changes in the parameters. The models were verified using the R^2^ for comparison of the actual data observed in the experiment with the data estimated in the model (Table 3). The kinetic models showed a significant correlation between the empirical data and the model estimates. This means that the models could successfully evaluate quality changes in processed cheese. The models did not show statistical significance of the following elements: loss modulus (G′), loss tangent (tgδ), difference in chroma (ΔC), red–green space a*, colour, consistency.

**Table 3:** Kinetic models of the analysed quality parameters

Parameter	R^2^	Model
Water activity	89.58	$Aw = \left[ {Aw_{0}^{ - 1} - 8.95 \times 10^{10} \exp \left( {\frac{ - 79.31}{8.31T} } \right)t} \right]^{ - 1}$
Acidity (pH)	99.71	$pH = \left[ {pH_{0}^{ - 1} - 8.78 \times 10^{14} \exp \left( {\frac{ - 97.63}{8.31T}} \right)t} \right]^{ - 1}$
Lightness L*	91.54	$L^{*} = L_{0}^{*} - 1.61 \times 10^{30} \exp \left( {\frac{ - 190.49}{8.31T}} \right){\text{t}}$
Redness a*	91.32	$a^{*} = a_{0}^{*} - 2.67 \times 10^{30} \exp \left( {\frac{ - 132.40}{8.31T}} \right){\text{t}}$
Yellowness b*	99.39	$b^{*} = \left[ {b^{*}_{0} \exp ( - 3.52 \times 10^{28} \exp \left( {\frac{ - 173.22}{8.31T} } \right)t} \right]$
Difference in colour ΔE	96.41	$\Delta {\text{E}} = \Delta {\text{E}}_{0} - 3.48 \times 10^{12} \exp \left( {\frac{ - 78.70}{8.31T}} \right){\text{t}}$
Difference in chroma ΔC	72.60	$\Delta {\text{C}} = \Delta {\text{C}}_{0} - 2.59 \times 10^{3} \exp \left( {\frac{ - 29.69}{8.31T}} \right){\text{t}}$
Dynamic viscosity η	85.74	$\upeta =\upeta_{0} - 1.09 \times 10^{7} \exp \left( {\frac{ - 402.80}{8.31T}} \right){\text{t}}$
Elastic modulus G′	99.20	$G^{{\prime }} = \left[ {G_{0}^{{{\prime } - 1}} - 2.48 \times 10^{109} \exp \left( {\frac{ - 621.79}{8.31T} } \right)t} \right]^{ - 1}$
Loss modulus G″	89.24	$G^{{\prime \prime }} = G_{0}^{{\prime \prime }} - 8.27 \times 10^{57} \exp \left( {\frac{ - 339.13}{8.31T}} \right){\text{t}}$
Loss tangent tgδ	84.41	${\text{tg}}\updelta = \left[ {{\text{tg}}\updelta_{0} \exp ( - 4.24 \times 10^{21} \exp \left( {\frac{ - 132.64}{8.31T} } \right)t} \right]$
Hardness	64.91	$Tw = {\text{Tw}}_{0} - 4.54 \times 10^{9} \exp \left( {\frac{ - 44.15}{8.31T}} \right){\text{t}}$
Spreadability	86.95	$Sm = \left[ {Sm_{0}^{ - 1} - 5.78 \times 10^{12} \exp \left( {\frac{ - 85.22}{8.31T} } \right)t} \right]^{ - 1}$
Viscosity index	76.52	$Lp = \left[ {Lp_{0}^{ - 1} - 5.02 \times 10^{14} \exp \left( {\frac{ - 95.47}{8.31T} } \right)t} \right]^{ - 1}$
Adhesiveness	92.25	$Prz = \left[ {Prz_{0}^{ - 1} - 1.54 \times 10^{11} \exp \left( {\frac{ - 76.29}{8.31T} } \right)t} \right]^{ - 1}$
Colour	70.13	$Br = {\text{Br}}_{0} - 1.34 \times 10^{24} \exp \left( {\frac{ - 154.71}{8.31T}} \right){\text{t}}$
Consistency	99.08	$K = {\text{K}}_{0} - 1.55 \times 10^{51} \exp \left( {\frac{ - 300.14}{8.31T}} \right){\text{t}}$
Smell	88.11	$Z = {\text{Z}}_{0} - 5.44 \times 10^{42} \exp \left( {\frac{ - 243.87}{8.31T}} \right){\text{t}}$
Taste	97.19	$S = {\text{S}}_{0} - 1.72 \times 10^{9} \exp \left( {\frac{ - 111.45}{8.31T}} \right){\text{t}}$
Overall desirability	83.35	$Op = {\text{Op}}_{0} - 1.20 \times 10^{41} \exp \left( {\frac{ - 236.29}{8.31T}} \right){\text{t}}$

The temperature in shop coolers significantly affects the deterioration of spreadable processed cheese during storage. The producer’s own research showed that the temperature in coolers was often higher than the maximum permissible temperature for this product, which may have accelerated its deterioration. Moreover, the manufacturer can only recommend the range of temperatures in a shop cooler and increase risk awareness but has no enforcement instruments.

The analysis showed that storage at 8 °C did not significantly affect the finished product quality and that the storage time was the only significant factor. On the contrary, both the storage time and temperature equally influenced changes in the quality parameters in the samples stored at 20 and 30 °C. There were changes in the following quality parameters: taste, smell and consistency. These changes were the most typical of this product (Christensen et al., 2003; Mortensen et al., 2004). There were similar changes, especially in consistency, observed in other studies. They were chiefly caused by the hydrolysis of polyphosphates contained in emulsifying salts and from the interaction between proteins and other molecules (Buňka et al., 2012).

Researchers often indicate zero- and first-order reactions as the most common in food, whereas second-order reactions are not frequently observed (Boekel, 2008). Our analyses confirmed these findings as most of the parameters were characterised by zero-order reactions. There were second-order reactions only in a few cases.

The changes in quality parameters during storage were typical of processed cheese, as described in available literature (BK Giulini GmbH-BU-FOOD/BL-DAIRY, 2012; Mortensen et al., 2004; Plaza-Rodriguez et al., 2015). Changes in the colour of processed cheese may have been caused by the high lactose content, conditions of production and composition of stirred-curd Cheddar cheese in the recipe for processed cheese (Piergiovanni et al., 1989). However, relationships between the increase in the rate of reaction and temperature, leading to intensified reactivity between the sugar and the amino group, may cause colour changes, which are also commonly observed in other food products (O’brien et al., 1998). The rheological parameter was the most stable feature of processed Gouda cheese due to the highest activation energy. It may have been caused by stable pH or interaction between casein and whey proteins, which affected the rheological parameters of processed cheese (Mleko, 2001; Mleko and Foegeding, 2000). When we compare the activation energy of rheological parameters of heated Cheddar cheese (E_a_ = 141.1 kJ/mol), we can be conclude that processed Gouda cheese had thicker structure than Cheddar cheese (Tunick, 2010).

The kinetic models were based on the value of the activation energy and the chemical reaction rate constant. They enabled identification of the direction and rate of changes in the spreadable processed Gouda cheese during storage. The kinetic models showed a significant correlation between the empirical data and the data determined with the models. It was in line with the findings concerning many other food products. Kinetic models were effectively applied to evaluate the development of non-specific smell and change in the colour of UHT milk (Singh et al., 2009), fresh vegetables (Zanoni et al., 2007) and minced beef (Limbo et al., 2009).

**Fig. 2:**
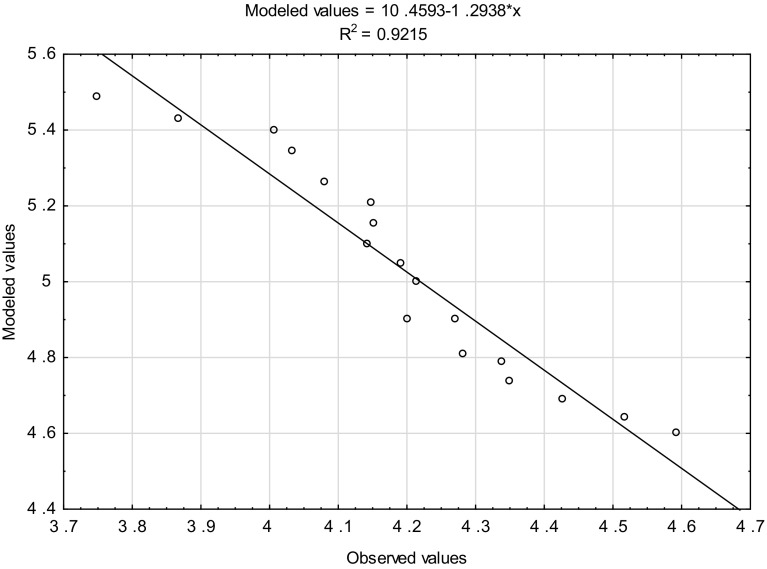
The correlation between the observed and modelled values referring to the general desirability of processed Gouda cheese

The experiment proved that the storage temperature and time significantly influenced deterioration of the quality of processed cheese. The activation energy values and the chemical reaction rate constants were used to prepare the kinetic models which enabled estimation of the trend and rate of changes in cheese quality during storage. The models showed that some quality parameters which are important for safety and consumers, i.e. hardness, water activity, pH and colour, had relatively low activation energy values (E_a_ = 29–97 kJ/mol). This means that these features exhibit high susceptibility to storage conditions. The highest values of activation energy were noted for the rheological parameters (E_a_ = 339–621 kJ/mol) and sensory properties, such as consistency, smell and overall desirability (E_a_ = 111–300 kJ/mol). These parameters were characterised by relatively high stability. Figure 2 shows an example diagram of the correlation between observed and model values referring to overall desirability. The empirical data are very well fitted with the model data (R^2^ = 0.9215). The Q parameter can be successfully used to predict the percentage loss of the quality of processed cheese resulting from the storage time and temperature.

## References

[CR1] Bao Y, Luo Y, Zhang Y, Shen Y, Shen H (2013). Application of the global index method to predict the quality deterioration of blunt-snout bream (*Megalobrama amblycephala*) during chilled storage. Food Sci. Biotechnol..

[CR2] Benítez S, Chiumenti M, Sepulcre F, Achaerandio I, Pujolá M (2012). Modeling the effect of storage temperature on the respiration rate and texture of fresh cut pineapple. J. Food Eng..

[CR3] BK Giulini GmbH-BU-FOOD/BL-DAIRY. Conference Materials. Processed cheese quality defects: rheological problems, crystallization, discoloration and microbiology (2012)

[CR4] Boekel M (2008). Kinetic Modeling of Food Quality: A Critical Review. Compr. Rev. Food Sci. Food Saf..

[CR5] Brosio E, Gianferri R. Low-resolution NMR—an analytical tool in foods characterization and traceability: basic NMR in foods characterization. Brosio, E. Reseach Signpost, Karela, India. pp. 233–237 (2009)

[CR6] Buňka F, Stetina J, Hrabe J (2008). The effect of storage temperature and time on the consistency and color of sterilized cheese. Eur. Food Res. Technol..

[CR7] Buňka F, Dudova L, Weiserova E, Kuchar D, Michalek J, Slavikova S, Kracmar S (2012). The effect of different ternary mixtures of sodium phosphates on hardness of processed cheese spreads. Int. J. Food Sci. Technol..

[CR8] Bunkar DS, Jha A, Mahajan A (2014). UnnikrishnanVS. Kinetics of changes in shelf life parameters during storage of pearl millet based kheer mix and development of a shelf life prediction model. J. Food Sci. Technol..

[CR9] Calligaris S, Manzocco L, Conte LS, Nicolia MC (2004). Application of a modified Arrhenius equation for the evaluation of oxidation rate of sunflower oil at subzero temperatures. J. Food Sci..

[CR10] Christensen J, Povlsen VT, Sorensen J (2003). Application of fluorescence spectroscopy and chemometrics in the evaluation of processed cheese during storage. J. Dairy Sci..

[CR11] Dong WL, Xiao W, Li DP, Sun ZH (2015). Prediction of quality and storage period of soybean in small packages under different temperature. Appl. Mech. Mater..

[CR12] Honga H, Luoa Y, Zhua S, Shenb H (2012). Application of the general stability index method to predict quality deterioration in bighead carp (*Aristichthys nobilis*) heads during storage at different temperatures. J. Food Eng..

[CR13] Kapoor R, Metzger LE (2008). Process cheese: scientific and technological aspects—a review. Compr. Rev. Food Sci..

[CR14] Keklik NM, Işikli ND, Sur EB (2017). Estimation of the shelf life of pezik pickles using Weibull hazard analysis. Food Sci. Technol..

[CR15] Limbo S, Torri L, Franzetti L, Casiraghi E (2009). Evaluation and predictive modeling of shelf life of minced beef stored in high-oxygen modified atmosphere packaging at different temperatures. Meat Sci..

[CR16] Mai Nga, Huynh Van (2017). Kinetics of Quality Changes of Pangasius Fillets at Stable and Dynamic Temperatures, Simulating Downstream Cold Chain Conditions. Journal of Food Quality.

[CR17] Mleko S (2001). High-pH gelation of whey protein isolate. Int. J. Food Sci. Technol..

[CR18] Mleko S, Foegeding EA (2000). Physical properties of rennet casein gels and processed cheese analogs containing whey proteins. Milchwissenschaft..

[CR19] Mortensen G, Bertelsen G, Mortensen BK, Stapelfeldt H (2004). Light-introduced changes in packaged cheeses—a review. Int. Dairy J..

[CR20] O'Brien John, Nursten Harry E., Crabbe M. James C., Ames Jennifer M. (1998). The Maillard Reaction in Foods and Medicine.

[CR21] Piergiovanni L, De Noni I, Fava P, Schiraldi A (1989). Nonenzymatic browning in processed cheeses. Kinetics of the Maillard reaction during processing and storage. Int. J. Food Sci..

[CR22] Plaza-Rodriguez C, Thoens C, Falenski A, Weiser AA, Appel B, Koesbohrer A, Filter M (2015). A strategy to establish Food Safety Model Repositories. Int. J. Food Micro..

[CR23] Singh RRB, Ruhil AP, Jain DK, Patel AA, Patil GR (2009). Prediction of sensory quality of UHT milk—a comparison of kinetic and neural network approaches. J. Food Eng..

[CR24] Spiess W, Boehme, Wolf (1997). Quality changes during distribution of deep-frozen and chilled foods. Food Storage Stability.

[CR25] Stangierski J, Rezler R, Leśnierowski G (2014). Analysis of the effect of heating on rheological attributes of washed mechanically recovered chicken meat modified with transglutaminase. J. Food Eng..

[CR26] Sui X, Yap PY, Zhou W (2015). Anthocyanins during baking: Their degradation kinetics and impacts on color and antioxidant capacity of bread. Food Bioprocess Technol..

[CR27] Sunesen LO, Lund P, Sorensen J, Holmer G (2002). Development of volatile compounds in processed cheese during storage. Lebensm.-Wiss.-Technol..

[CR28] Tang X, Sun X, Wu VCH, Xie J, Pan Y, Zhao Y, Malakar PK (2013). Predicting shelf-life of chilled pork sold in China. Food Control..

[CR29] Tsironi T, Salapa I, Taukis P (2009). Shelf life modelling of osmotically treated chilled gilthead seabream fillets. Innov. Food Sci. Emerg. Technol..

[CR30] Tunick MH (2010). Activation energy measurements in rheological analysis of cheese. Int. Dairy J..

[CR31] Varga L (2005). Use of a long-chain polyphosphate mixture for shelf-life extension of processed cheese spreads. A. Alim..

[CR32] Weiss D, Kaczmarek A, Stangierski J (2015). Applicability of bacterial growth models in spreadable processed cheese. Acta. Sci. Pol. Technol. Aliment..

[CR33] Zanoni B, Lavelli V, Ambrosoli R, Garavaglia L, Minati J, Pagliarini E (2007). A model to predict shelf-life in air and darkness of cut, ready-to-use, fresh carrots under both isothermal and non-isothermal conditions. J. Food Eng..

[CR34] Zhi NN, Zong K, Thakur K, Qu J, Shi JJ, Yang JL, Yao J, Wei ZJ (2018). Development of a dynamic prediction model for shelf-life evaluation of yogurt by using physicochemical, microbiological and sensory parameters. CyTA J. Food..

